# Micro-continuum approach for mineral precipitation

**DOI:** 10.1038/s41598-021-82807-y

**Published:** 2021-02-10

**Authors:** Fengchang Yang, Andrew G. Stack, Vitalii Starchenko

**Affiliations:** Chemical Sciences Division, Oak Ridge National Laboratory, 1 Bethel Valley Rd., Oak Ridge, TN 37831 USA

**Keywords:** Geochemistry, Chemical physics

## Abstract

Rates and extents of mineral precipitation in porous media are difficult to predict, in part because laboratory experiments are problematic. It is similarly challenging to implement numerical methods that model this process due to the need to dynamically evolve the interface of solid material. We developed a multiphase solver that implements a micro-continuum simulation approach based on the Darcy–Brinkman–Stokes equation to study mineral precipitation. We used the volume-of-fluid technique in sharp interface implementation to capture the propagation of the solid mineral surface. Additionally, we utilize an adaptive mesh refinement method to improve the resolution of near interface simulation domain dynamically. The developed solver was validated against both analytical solution and Arbitrary Lagrangian–Eulerian approach to ensure its accuracy on simulating the propagation of the solid interface. The precipitation of barite (BaSO_4_) was chosen as a model system to test the solver using variety of simulation parameters: different geometrical constraints, flow conditions, reaction rate and ion diffusion. The growth of a single barite crystal was simulated to demonstrate the solver’s capability to capture the crystal face specific directional growth.

## Introduction

Mineral precipitation commonly occurs in nature and plays an important role in many energy-related applications. For instance, minerals precipitate as scale in the pore structure near wellbores and significantly reduce the permeability of the porous formation^1^. This can lead to a rapid decrease in production and cause financial loss^2^. The need to predict the dynamic properties of such systems has resulted in questions about the fundamental mechanisms of mineral precipitation in pores^3^. Additionally, there is still a discrepancy between laboratory molecular scale findings and large-scale observations^4^. To address this discrepancy, modeling methods at the pore scale started gaining interest recently due to the capability of capturing reactive and nonreactive species transport, effects of pore topology, and interface chemical reaction within the same approach, which typically is difficult to observe in experiments^5^.

**Figure 1:**
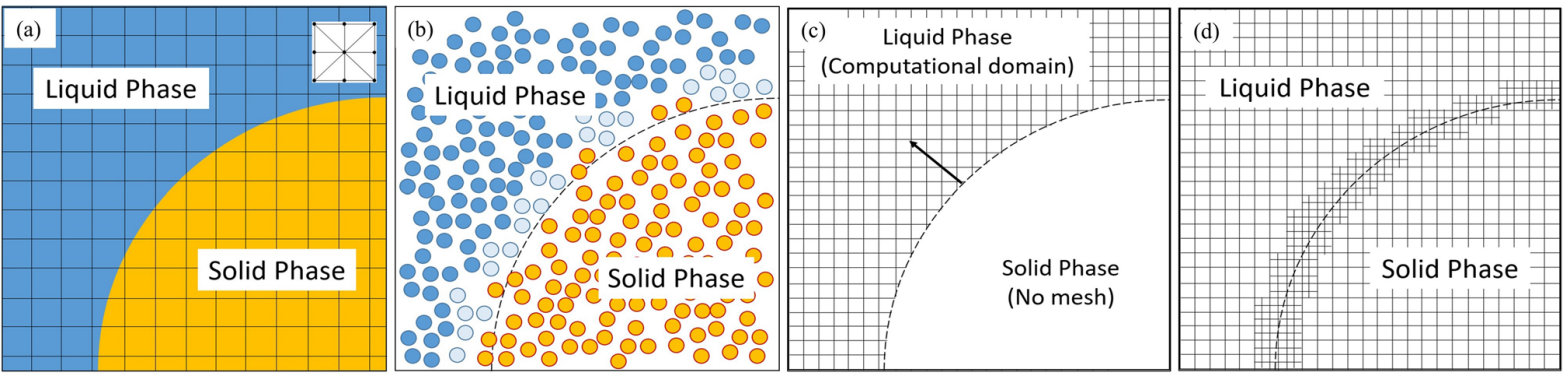
Schematic representation of the fluid–solid interface using different methods for pore-scale modeling of mineral dissolution or precipitation process (**a**) LBM (**b**) SPH (**c**) ALE (**d**) DBS.

Over the last decade several numerical approaches have been developed to model mineral dissolution and precipitation processes. Some of the commonly used approaches rely on different numerical formulation such as Lattice Boltzmann method (LBM), smoothed particle hydrodynamics (SPH), computational fluid dynamics (CFD). The LBM originated from gas kinetic theory and computes the statistical functions for phenomena such as fluid flow, species transport, etc*.* LBM typically divides the computational domain into uniformly distributed lattices, which are populated by a set of discrete velocity vectors^6^. Applied to the problem of reactions on mineral–water interface LBM grid cells need to be marked as belonging to the liquid and solid phase respectively. Figure 1a illustrates the assignment of the grid points: “blue area”—liquid, “yellow area”—solid. Kang and colleagues studied a wide range of dissolution and precipitation problems related to reacting flow in pores using LBM models^7–9^. Later, using a similar model, Chen et al. found that the precipitation of a secondary solid phase inhibited the dissolution of the primary solid phase^10^ due to the surface coverage. Szymczak and Ladd used LBM to model fluid flow in fractures in combination with random walk algorithm for transport of dissolved species which was applied to study the wormhole formation phenomenon in natural rock^11,12^. Due to the nature of LBM algorithm that is linked to the regular Cartesian grid, it is easy to implement, and its application shows a great potential at handling mineral dissolution/precipitation at mesoscale. However, LBM models are accompanied by a relatively small time step compared to other methods and in turn requires more computational resources^13^. Moreover, in order to capture a multiphase interface, uniformly distributed lattices may require significant computational resources, which sometimes can be only obtained at Leadership Computing Center level (e.g., OLCF, ALCF)^14^.

Due to the complex geometry of mineral–water interfaces and need to reduce computational load, meshless particle-based methods were adapted to model dissolution and precipitation processes. As an example, a mineral–water interface representation within smoothed particle hydrodynamics method is shown on Fig. 1b. This method represents a physical domain by discretizing it into small particles and solving both the flow and transport equations. Then the fluid flow and species transport are tracked by each individual Lagrangian particle motion. The original SPH was introduced by Lucy^15^ and Gingold^16^ for simulating the fluid dynamics related to astrophysical areas. Since 2007, Tartakovsky et al*.* developed a model using SPH method to study the reactive transport and mineral precipitation in fractured and porous materials^17,18^. The particle-based methods do not rely on a mesh and are good at dealing with evolving geometries. However, the identification and tracking of liquid/solid interface is not trivial in SPH and requires a coupling of additional methods.

As an alternative, fluid flow and species transport equations can be solved using finite volume (FVM), finite element (FEM) or finite difference (FDM) methods^19^. The use of unstructured meshes (including complex geometries with curved boundaries) allows one to resolve the interface and to reduce need for computational resources. Similarly, to model processes that lead to growing (or dissolving) phases it is necessary to track the interface between those phases. The most popular approaches that allow one to model and track interfaces during multiphase CFD modeling include Volume of Fluid (VOF)^20^, Level Set (LS)^21^, and Arbitrary Lagrangian–Eulerian (ALE)^22^ methods. A boundary can be captured precisely using the ALE approach, in which the surface mesh is moved continuously following the phase evolution. However, it is hard to implement and topological changes or remeshing are needed to maintain the mesh quality after significant deformation of the initial shape. The Level Set method solves an extra level-set equation, which allows the location and curvature of the interface to evolve by advecting and re-initializing the level-set function. For instance, Jones and Detwiler have used this approach to fit the experimental observation of calcium carbonate precipitation in fractures^23^. Although the LS method allows one to capture the curvature of interface more accurately, it typically requires more computational resources and can sometimes lead to non-conservative solutions if no special treatment is implemented.

Recently a micro-continuum Darcy-Brinkman-Stokes (DBS) approach has been successfully applied to model mineral dissolution^24^. The DBS model solves a single set of governing equations for liquid/solid/porous phases similar to fictitious domain method in which a penalty source term is used to switch the behavior of governing equation according to the volume fraction of certain phase within the computational cell^25^. Such treatment of phases allows one to include in the simulations a flexible geometry of different phases and the reactions at the interface between phases is captured through an immersed boundary method without explicit tracking. To include the gas phase production during solid matrix dissolution, which requires surface curvature to take into account surface tension, Soulaine et al*.* coupled the DBS approach with the volume of solid (VOS) interface tracking method^26^. They demonstrated that the approach captures dynamics of all three phases by comparing modeling with a microfluidic experiment. Although the DBS method has shown a great potential to model mineral phase change with interface evolution, only very limited previous research has been done to study mineral precipitation to the authors’ best knowledge. Recently, a benchmark study on the performance and robustness of different approaches (DBS, LBM, ALE, etc*.*) in respect to a single particle dissolution problem has shown that all methods mentioned above can achieve a good agreement^27^. However, one should take into account strength and weakness of each approach before applying it to a new problem.

Other methods such as the phase field modeling can be also used to simulate mineral precipitation from solutes^28^. In contrast to the described above methods, the evolution of solid precipitate in phase field approach is modeled using an order parameter, which follows a separate differential equation and substitutes the surface reaction rate equation by the minimization of the free energy difference between solid and liquid phases^29^. The solid–fluid interface in the phase field approach is represented by the diffuse boundary and is controlled by a parameter that is a part of the differential equation for the order parameter. Although early phase field models for mineral precipitation were developed and coupled with the diffusion equation^30^ or just as a minimization of the free energy functional^31^, recent development includes coupling with the Navier–Stokes equation^32^.

Due to the complexity of the mineral precipitation process within the porous medium, there is a great interest in improving the existing models. Unlike previous DBS studies (which mostly focused on the dissolution process), in this paper, we explore the possibility of using the DBS framework with volume of solid (VOS) that implements a sharp transition from liquid to solid for studying mineral precipitation at the micro-continuum scale. Our choice of DBS is due to its flexibility in dynamically capturing appearing and disappearing phases. To model the interface more accurately, we use adaptive mesh refinement method which was added within the newly developed solver to locally refine the mesh near the liquid–solid interface. In addition, a criterion for propagation of a sharp interface was introduced to limit the diffusion effect of the native DBS scheme, which may lead to inaccurate representation. Moreover, since precipitation of crystals typically shows different face-specific growth rates^33^, an extra directional growth method was developed to improve the capability of the solver on modeling the realistic crystal growth. Due to the importance of the mineral barite (BaSO_4_) in the scale formation^34^, we applied this solver to model several cases of barite crystal growth precipitation.

This manuscript starts with the mathematical details of the multiphase micro-continuum model and the sub-grid models for reactions and interface. Further, we present the adapted mesh approach for more robust capture of interface propagation. After that, the developed solver is validated against both analytical solution and an established ALE solver—dissolFoam^35^ that is referred in the rest of the manuscript as the ALE solver. Then, the effect of geometrical constraint, flow condition, reaction rate and ion diffusion on barite precipitation are presented followed by modeling of a single crystal growth. Finally, the directional growth of a single barite crystal with different facets, with variate growth rates, was simulated to show the potential of this solver in modeling a wide array of crystallization problems.

## Method

In this section, we introduce the underlying mathematical model for the pore-scale flow and species transport through a porous medium. To represent both solid and liquid phases in the simulations we used the volume of solid (VOS) method (similar to volume of fluid method) to include effects of solid phase on the fluid flow. The simulation domain is discretized into computational cells of a control volume, ${V}_{c}$, which contain two scalar fields ${\varepsilon }_{s}$ and ${\varepsilon }_{f}$. The scalar fields represent the volume fractions of both solid (*ε*_*s*_) and liquid phases (*ε*_*f*_) within the cell. The following correlation holds for each computational cell,

1 $${\varepsilon }_{f}+{\varepsilon }_{s}=1.$$

Therefore, the cell that is fully occupied by liquid results in ${\varepsilon }_{f}=1$ and ${\varepsilon }_{s}=0$. The opposite holds for the cell fully occupied by solid phase. Such a method represents the liquid–solid interface through a diffuse interface model (interface exists in region where $0<{\varepsilon }_{f}, {\varepsilon }_{s}<1$), which does not explicitly capture a sharp interface. Instead, the interface could exist within one or multiple layers of cells depending on the implementation.

In this study, we follow the fictitious domain method for modeling the flow within a diffuse interface layer^24,25,36,37^ that is represented analogously to a porous region. The flow of the dilute solution is governed by one form of the Navier–Stokes/Brinkman equation,

2 $${\rho }_{f}\left(\frac{\partial \stackrel{-}{{\varvec{u}}}}{\partial t}+\left(\stackrel{-}{{\varvec{u}}}\cdot \nabla \right)\stackrel{-}{{\varvec{u}}}\right)=-\nabla \stackrel{-}{p}+{\mu }_{f}{\nabla }^{2}\stackrel{-}{{\varvec{u}}}+{S}_{p},$$

where $\stackrel{-}{{\varvec{u}}}$ is the superficial velocity for each computational cell, $\stackrel{-}{{\varvec{u}}}=\frac{1}{{V}_{c}}{\int }_{{V}_{f}}{\varvec{u}}dV$, $\stackrel{-}{p}$ is the intrinsic phase averaged pressure, $\stackrel{-}{p}=\frac{1}{{V}_{c}}{\int }_{{V}_{f}}pdV$, ${\mu }_{f}$ is the dynamic viscosity of solution. The non-slip boundary condition on liquid–solid interface $\Gamma$ for the solid phase in introduced via a penalization term, ${S}_{p}$, which vanishes in the liquid phase and forces velocity toward zero in the solid phase. Different form of this penalization term could be used according to the applications. Specifically, in this study, we follow the well-known Kozeny-Carman relation^36^ to estimate the local permeability in a computational cell that is represented by the precipitate,

3 $${S}_{p}=-\frac{{\left(1-{\varepsilon }_{f}\right)}^{2}}{K{\varepsilon }_{f}^{3}}\stackrel{-}{{\varvec{u}}},$$

where $K$ is the combined permeability constant. If $K\to \infty$, this penalty term vanishes within the pure liquid region and the governing equation reduces to the standard incompressible Navier–Stokes equation. If $K\to 0$, the governing equation enforces a non-slip boundary condition at interface and freeze the motion of solid phase. However, if a porous region exists, a moderate $K$ value can be chosen to mimic the Brinkman equation for porous medium^25^. In this study, due to the relatively thin porous (or interface) region and slow growth rate of solid phase, we chose a small $K$ value (~ 10^–20^) to help implementing a non-slip boundary condition at interface.

During the precipitation process, the solid phase evolves along with the precipitation happening at the liquid–solid interface. By assuming only one type of mineral is precipitating, the solid volume fraction in a local cell can be found through,

4 $$\frac{\partial {\varepsilon }_{s}{\rho }_{s}}{\partial t}=-{M}_{s}\dot{R},$$

where $\dot{R}$ is the volume-based reaction rate ($\frac{\text{mol}}{{\text{m}}^{3}\; \text{s}}$) and ${M}_{s}$ is the molar mass for solid phase. Naturally, the continuity equation for the fluid phase reads

5 $$\frac{\partial {\varepsilon }_{f}{\rho }_{f}}{\partial t}+\nabla \cdot \left({\rho }_{f}\stackrel{-}{{\varvec{u}}}\right)={M}_{s}\dot{R}.$$

The transport of chemical species is governed by the diffusion–advection equation with source terms for including the chemical reactions,

6 $$\frac{\partial {{\varepsilon }_{f}C}_{i}}{\partial t}+\nabla \cdot \left(\stackrel{-}{{\varvec{u}}}{C}_{i}\right)=\nabla \cdot \left({D}_{eff}\nabla {C}_{i}\right)-\dot{R},$$

where ${D}_{eff}$ is the effective diffusion coefficient in form of ${D}_{eff}={\varepsilon }_{f}^{2}D$ and $D$ is the bulk diffusion coefficient ($\frac{{\text{m}}^{2}}{\text{s}}$). The precipitation of a mineral within eligible computational cell is controlled by the correlation obtained from previous experiments in closed system reactors^38^. In the current model we assume a precipitation of a sparingly-soluble 1:1 salt—barite (BaSO_4_), where one ion of Ba^2+^ consumed corresponds to one anion of SO_4_^2−^ also consumed. Thus, using the principle of electroneutrality we can assume that the averaged local concentration of both ions in a simulation cell is the same at the pore-scale and we can consider a transport of only one species. Of course, this assumption doesn’t hold if the transport is considered in nano-porous material or within the interfacial region if there is a large surface charge. However, we consider this case to be out of the scope of the current model. Although more complex expressions can be derived (e.g., Refs.^39,40^), for this initial work we use the surface-controlled dissolution and precipitation rates per unit area fitted in the form of an empirical rate law:

7 $$\dot{r}=k{\left(\Omega -1\right)}^{n},$$

where *k* ($\frac{\text{mol}}{{\text{m}}^{2} \;\text{s}}$) is a fitted rate constant from experiments, the dimensionless $\Omega =\frac{{a}_{{Ba}^{2+} }{a}_{{SO}_{4}^{2-} }}{{K}_{barite}}$ is the saturation state of the liquid with respect to barite, ${a}_{i}$ represents the activity of corresponding aqueous ion, ${K}_{barite}$ is the equilibrium constant for barite in liquid, $n$ is the power for the reaction ($n$ = 1 for linear growth, $n$ = 2 for spiral growth). Note, our current model introduces the precipitation as a reaction source term to simplify the complexity of the model. However, for high saturation index solution, the nucleation process could potentially play a very important role in the precipitation due to either heterogeneous or homogenous nucleation, which should be considered in the future studies. In this study, we are focusing more on moderate saturation index solution, hence the simplified uniform precipitation was applied.

However, unlike the ALE method, no embedded boundaries (Fig. 1c) were used to represent the liquid–solid interface in the fictitious domain method. In this study, an immersed boundary method (Fig. 1d) was used to represent the interface and calculate the volume-based reaction rate in the computational cell, i.e.,

8 $$\dot{R}=\frac{1}{{V}_{c}}{\int }_{{A}_{ls}}\dot{r}dA\approx {a}_{v}\dot{r},$$

where ${a}_{v}$ ($\frac{{\text{m}}^{2}}{{\text{m}}^{3}}$) is the area density in the eligible computation cell that is undergoing precipitation. By mapping surface area to volume-based density, this approximation is similar to the assumption of sharp transition from liquid phase to solid phase by Soulaine et al.^24^. Sharp transition assumption restricts the propagation of the solid phase until the current cell is fully filled with a precipitate. Therefore, the area density relates the gradient of porosity with the surface area in certain control volume ${a}_{v}=\Vert \nabla {\varepsilon }_{f}\Vert$. This approach ensures no interface exists in pure solid/liquid regions. However, a diffusive interface may emerge due to the nature of the DBS model, which can spread across several layers of computational cells. To limit this effect and ensure the sharpness of the interface, a criterion of the solid volume fraction, ${\varepsilon }_{s,c}$, was imposed to limit the distance of the diffuse interface to one layer of computational cell. In another words, a computational cell was only enabled for solid growth when a nearby cell with solid volume fraction higher than the criterion (i.e., ${\varepsilon }_{s,c}$) exists (see “Model validation” section for details). For results comparison we used nondimensional time, ${t}^{*}=t/\tau$, which was scaled to the diffusion time defined as $\tau ={d}^{2}/D$ with $d$ as a sphere diameter.

**Figure 2:**
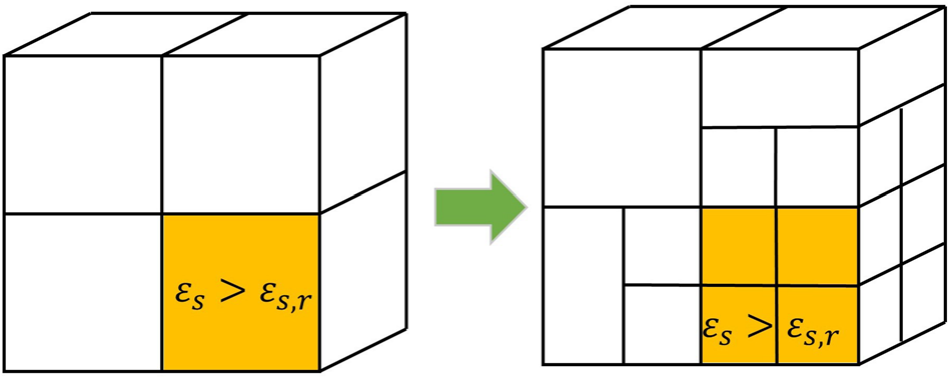
Local computational cell refinement is based on the volume fraction of solid phase.

One limitation of this combined fictitious domain method and diffuse interface model is that the interface is typically presented through a gradient of the volume fraction, which relates to the local mesh size near the interface. It is computationally expensive to reduce the mesh size in the whole computational domain in which the solid phase only appears in some regions. To address this problem and capture the interface more accurately, we employed an adaptive mesh method^41^ to refine the mesh automatically near the tracked interface (see Fig. 2). This method splits the computational cell when the volume fraction of solid bypass certain threshold, ${\varepsilon }_{s,r}$, and merges them when all nearby cells are occupied by the solid phase. In this study, the volume fraction of solid, ${\varepsilon }_{s}>0.01$, was used to automatically refined the mesh near solid–fluid interface.

Recently, a study showed that the dissolution of minerals such as gypsum (CaSO_4_·2H_2_O) could be affected by the additional hindrance on the ion diffusion from the interaction between neighboring ion clouds^42^. In the current study, we also introduced an activity correction to ion diffusion to investigate its impact on the barite precipitation process

9 $$D\left(c\right)={D}_{0}\left(1+\frac{d ln\gamma }{d lnC}\right),$$

where $\gamma$ is the ion activity coefficient, ${D}_{0}$ is the bulk diffusion coefficient and $C$ is the ion concentration. Following the extended Debye-Hückel theory^43^, the activity coefficient can be linked to the barium concentration by a correlation,

10 $$ln{y}_{\pm }=-\frac{A\sqrt{\frac{C}{{C}_{sat}}}}{1+B\sqrt{\frac{C}{{C}_{sat}}}},$$

where ${C}_{sat}$ is the saturation concentration, A and B are coefficients related to the parameters in the extended Debye-Hückel theory.

Modeling growth of a single crystal is another challenging problem due to crystal face dependent reaction rates. In most past studies of mineral precipitation, the rate constant was maintained as isotropic across the whole simulation domain since the main goal was to model a volume averaged precipitate that does not resolve a shape of a single crystal^17,24,27^. In this study, we explore modeling a single crystal growth by applying the anisotropy of reaction rate as a function of crystallographic orientation. Different values of the reaction constant ${k}_{i}$ were assigned to the local cell by calculating its max similarity function between surface norm, ${\overrightarrow{n}}_{s}$, and prescript directional growth norms, ${\overrightarrow{n}}_{dir}$,

11 $${\cos}\left(\theta \right)=\frac{{{\overrightarrow{n}}_{s}\cdot \overrightarrow{n}}_{dir}}{| {{\overrightarrow{n}}_{s}} |{|\overrightarrow{n}}_{dir}|}.$$

The surface norm was obtained by calculating the normalized directional gradient of the solid phase VOS, ${\overrightarrow{n}}_{s}=\nabla {\varepsilon }_{s}$/|$\nabla {\varepsilon }_{s}|$. In another word, for each different ${\overrightarrow{n}}_{dir}$, its norm-based reaction constant ${k}_{i}$ was used for calculating precipitation if its $\text{cos}(\theta )$ is the max among all ${\overrightarrow{n}}_{dir}$ for certain surface. The goal of this model is to reproduce the anisotropy growth process observed in experiments while maintain the general shape of the crystal.

All above mathematical formulations were discretized using finite volume approach. The time step was controlled by setting Courant–Friedrichs–Lewy number smaller than 0.5. The solver accuracy was setting to 1e−8, which was small enough to ensure convergence. A mesh sensitivity study was performed to ensure the precipitation process was independent of the chosen mesh size by carefully changing the mesh size and comparing results deviation for all simulation cases. We implemented the model within the solver mpFoam using an open source CFD package OpenFOAM^44^. The temporal discretization was handled using Crank-Nicolson method, while the spatial discretization was handled using the default OpenFOAM method (Gauss linear). The details of the numerical method can be found in the User Guide of OpenFOAM. To perform the simulations, we utilized the CADES cluster at Oak Ridge National Laboratory and Lawrence Berkley National Lab’s NERSC supercomputer.

## Model validation

To show that the current solver is capable of simulating the fluid flow, species transport and precipitation processes, two separate validation cases were conducted. The first case was performed to validate the effectiveness of capturing moving interface and accuracy of sub-grid phase change model. This simulation was set up similar to the 1D non-linear diffusion validation problem for SPH model developed by Tartakovsky^17^. The solid phase was immersed in a supersaturated solution ($C\left(x,t=0\right)={C}_{0}$) and precipitation occurred only at the front surface of the solid phase. The total simulation domain had a length of $L=32\delta$ ($\delta$ is the unit length) and the solid phase occupied region from 0 to $\delta$. A prescribed concentration $C\left(L, t\right)={C}_{0}$ was imposed as a boundary condition for $x=L$ and the precipitation reaction at front surface follows a first order kinetic correlation, $D\nabla C\cdot {\varvec{n}}={k}_{l}\left({C}_{i}-{C}_{eq}\right)$, where ${k}_{l}$ is local reaction rate constant and ${C}_{eq}$ is the equilibrium solution concentration at solid–liquid interface. By assuming slow growth processes, no fluid flow, an approximate analytical form can be obtained from diffusion equation as follow,

12 $$S\left(t\right)=\left(\frac{D}{{k}_{l}}+L\right)-\sqrt{{\left(\frac{D}{{k}_{l}}+L-{S}_{0}\right)}^{2}-\frac{2D\left({C}_{0}-{C}_{eq}\right)t}{{\rho }_{m}}}.$$

**Figure 3:**
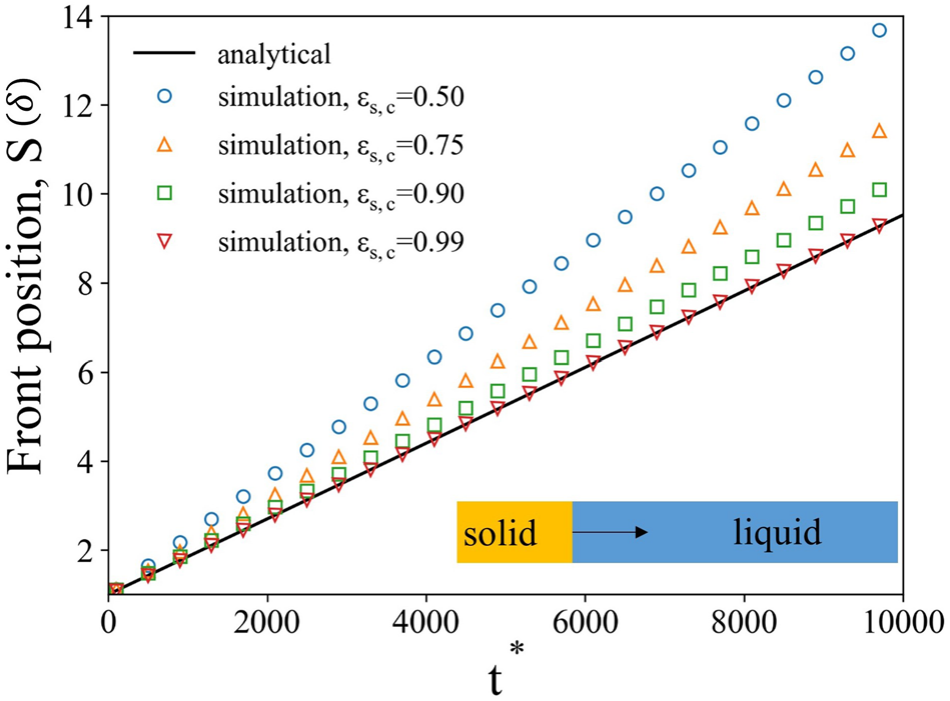
Position of liquid–solid interface as a function of nondimensional time obtained from mpFoam simulation with different ${\varepsilon }_{s,c}$ and analytical solution.

The other input parameters for simulation and analytical solution are ${k}_{l}$ = 0.001 $\frac{{\text{m}}}{{\text{s}}}$, $D$ = 1 ${\text{m}}^{2}/\text{s}$, $\frac{{C}_{0}}{{C}_{eq}}=8$, ${\rho }_{m}=16\frac{\text{kg}}{{\text{m}}^{3}}$. As mentioned in “Method” section solid interface propagates only when a cell has solid volume fraction higher than ${\varepsilon }_{s,c}$. To investigate the effect of this chosen criterion ${\varepsilon }_{s,c}$ on the solid interface propagation, we picked ${\varepsilon }_{s,c}$ = 0.5, 0.75, 0.9, and 0.99, and compared results with the analytical solution. Figure 3 shows the comparison of the simulation results from mpFoam and the analytical solution according to Eq. (9). As the results show, when ${\varepsilon }_{s,c}$ was reduced from 0.99 to 0.5, the front position S gradually deviated from the analytical solution. This deviation means the interface in the model became diffusive and didn’t satisfy the sharp interface analytical solution. In turn, the front of the solid phase accelerated and resulted in a porous solid, in which species transport is still possible. This scenario remains useful if modeled precipitate has, for example, nanoporous structure. In such cases an appropriate transport in porous region should be implemented. In contrast, if ${\varepsilon }_{s,c}$ was maintained at a value closed to 1 (${\varepsilon }_{s,c}$ = 0.99 case in Fig. 3), the current sub-grid phase change model can accurately calculate the amount of species precipitate and propagate the location of interface accordingly. Hence, in this study, as we were mainly focusing on the mineral formation of the barite crystal, a higher value of ${\varepsilon }_{s,c}$ (0.99) was used.

**Figure 4:**
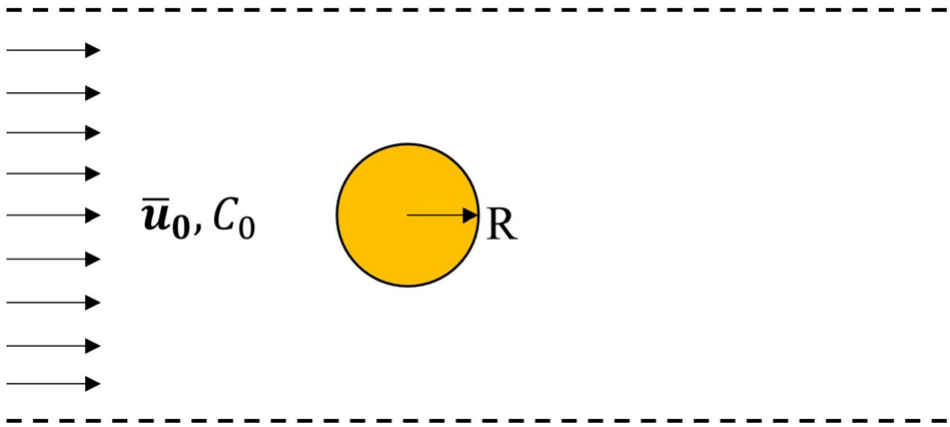
Schematics of validation case setup for both mpFoam and ALE solver.

Additionally, to take into account the fluid flow and convective transport of the species in solution, we performed a benchmark simulation of precipitation on a sphere. The dynamics of the solid precipitate interface was compared to the established ALE solver^35,42^. Figure 4 shows the setup of the simulation. A solid sphere of 100 $\upmu \text{m}$ radius was placed in a computational domain of dimension 2.1 mm $\times$ 1.5 mm $\times$ 1.5 mm. The sphere was located at 700 $\upmu \text{m}$ away from the left-side inlet and at the center of y–z plane. At the inlet, the solution flows at a speed of ${\stackrel{-}{u}}_{0}$ = 100 $\frac{\upmu \text{m}}{\text{s}}$ and has a concentration of reactant that corresponds to ${C}_{0}$ = 0.32 $\frac{\text{mol}}{{\text{m}}^{3}}$. We consider concentration of barium and sulfate ions to be equal in the simulation cell, as we explained in previous section. The saturation concentration for barium sulfate at room temperature is ${C}_{s}$ = 0.0105 $\frac{\text{mol}}{{\text{m}}^{3}}$. The precipitation reaction follows Eq. (7) and the reaction constant is taken from the barite precipitation study by Zhen-Wu et al. as $k$ = 1.479 $\times$ 10^–9^ $\frac{\text{mol}}{{\text{m}}^{2} \; \text{s}}$^38^. The viscosity of the solution is ${\mu }_{f}$ = 0.89 $\times$ 10^–6^ $\frac{{\text{m}}^{2}}{\text{s}}$ and diffusion coefficient for ions is $D$ = 1.4 $\times$ 10^–9^ $\frac{{\text{m}}^{2}}{\text{s}}$. The right-side outlet was set to open to a large reservoir (outlet boundary condition), while other boundaries were set as symmetry. In turn, barite precipitated on the surface of the sphere followed by continuous growth process.

**Figure 5:**
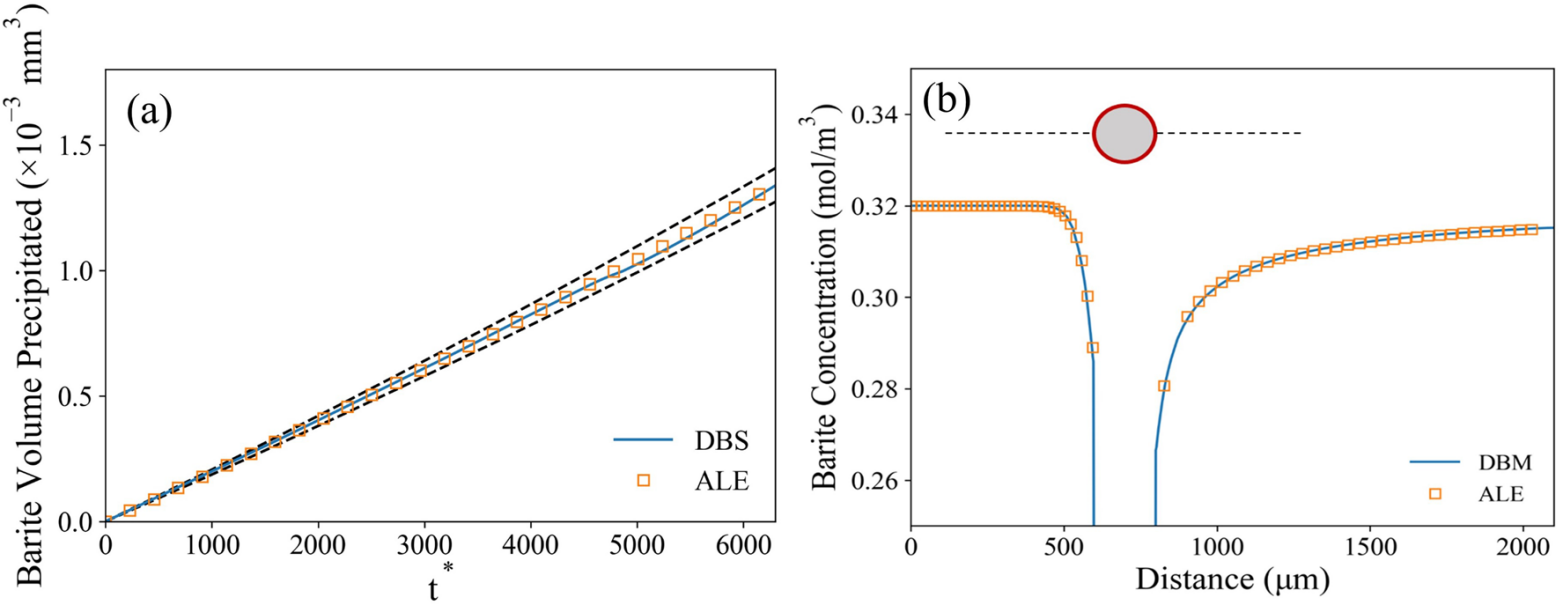
(**a**) Volume of precipitated barite as a function of nondimensional time obtained from mpFoam simulation and ALE solver (dash lines are 5% error line). (**b**) Reactive species concentration along the center line of the sphere from mpFoam and ALE solver.

Figure 5 compares the results from the benchmarking simulations conducted in both mpFoam and ALE solver. The mpFoam simulation results show good agreement with the ALE solver for both the total volume of precipitated barite (Fig. 5a) as well as the barium sulfate concentration distribution within the domain (Fig. 5b). Note, about 1% deviation still exist due to nature difference of interface treatment between DBS and ALE, but this has a minor impact on the overall results. The snapshots of liquid–solid interface from both solvers at different times are plotted in Figure S1 (see the Supporting Information). Compared to methods like ALE, the DBS model can handle the coalescence/separation of solid phase rather naturally. An extra case of two grains merge together due to precipitation was shown in the Figure S2 (see the Supporting Information).

The validation cases described in this section allow us to assume that the mpFoam solver can capture the interface propagation, fluid flow and chemical reaction accurately. We validate our model against analytical solution and other numerical method, however, further experimental verifications for different application may still be valuable considering the application scenario.

## Results and discussion

In this section we discuss the application of the solver to study the precipitation process of minerals (particularly, barite) at pore-scale. We investigated the impact of geometrical constrains and porosity on the precipitation and reactant transport at pore-scale. Then, the effects of various flow conditions, reaction rate and diffusion coefficient correction were explored to demonstrate the capability of the solver. The time in each cases was nondimensionalized by $\tau ={d}^{2}/D$ with $d$ as characteristic length, e.g., the sphere diameter in most cases of this study.

### Effect of geometrical constrain and porosity

**Figure 6:**
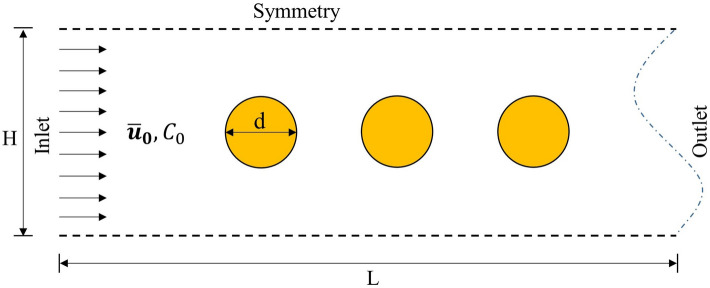
Schematic representation of a simulation setup for of reactive flow through porous medium.

To investigate the effects of porosity and geometrical constraints on the precipitation a 3D geometry that includes several spherical particles was constructed similar to the previous studies by Soulaine et al.^24^. The schematic representation of the simulation system is shown in Fig. 6. The height (*H*) and width (*W*) of the simulation domain were varied to adjust the porosity of the representative elementary volumes. The radius of spherical particles is R = 100 $\upmu \text{m}$ and the length of the simulation domain (*L*) is set as *L* = 3 mm. Four spherical particles were arranged along the centerline of the simulation domain in the x-direction along the flow direction. The distance between two particle centers was 200 $\upmu \text{m}$. The supersaturated solution of barium sulfate flowed into the simulation domain from the left side inlet at a constant speed of ${\stackrel{-}{u}}_{0}$ = 5 $\frac{\upmu \text{m}}{\text{s}}$. The saturation index, $\text{SI}=\text{log}(\Omega$), of reactive solution at the inlet is 3 (any higher an SI has resulted in homogenous nucleation in experiments^45^). The right side boundary was set as an outlet boundary condition. The other four sides of the simulation domain were set as symmetric boundary conditions to mimic a large porous medium in both *y* and *z*-directions. All other parameters regarding the barite precipitation are the same as the ones used in the validation case.

**Figure 7:**
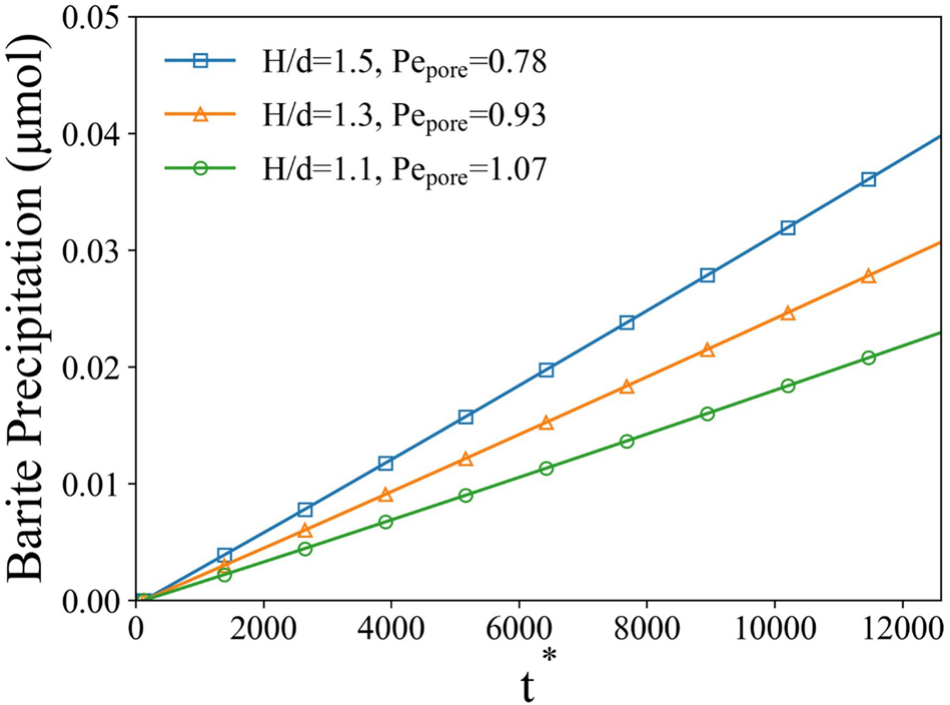
Mass of precipitated solid phase in whole domain as a function of nondimensional time for different geometrical constrains *H/d* and *Pe*.

To investigate the effect of geometrical constraints on the precipitation process, we gradually change both the ratios $H/d$ and $W/d$ simultaneously from 1.05 to 1.5 and keep the other inputs unchanged. Such constrains demonstrate the permeability/porosity changing (reduction of $H/d$) in porous materials that results in reduced transport of species. To demonstrate the results in terms of *Pe* number we introduce ${Pe}_{\text{pore}}=\frac{{\stackrel{-}{{\varvec{u}}}}_{0}{d}_{\text{pore}}}{D},$ where ${d}_{\text{pore}}$ is the hydraulic diameter of the pore. The total precipitated solid mass was obtained by integrating solid mass in each cell through the whole computational domain. It was observed that the total precipitated solid mass decreases as the simulation domain became narrower (see Fig. 7). The total amount of precipitated solid mass in the *H/d* = 1.1 ($W/d$ = 1.1) case was only about 55% of the solid mass in the *H/d* = 1.5 case. Another observation from the simulation results is that the precipitation follows a linear growth in all cases even though the geometrical constraint was imposed by shrinking the size of simulation domain.

**Figure 8:**
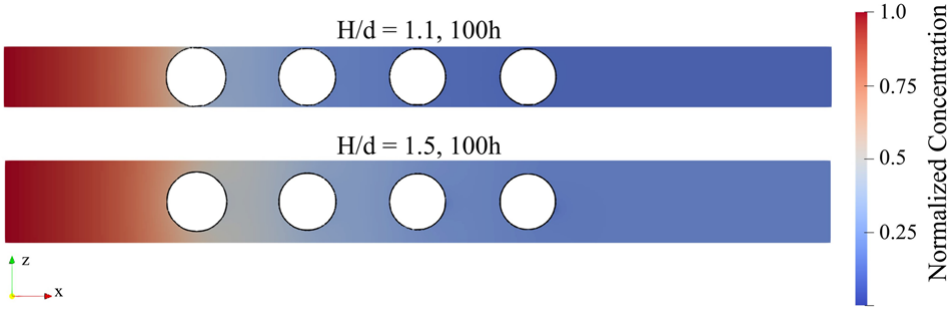
Normalized concentration distribution ($C/{C}_{0}$, central x–z plane) for different geometrical constraints case at 100 h simulation time (${t}^{*}$ = 12,600).

This difference in total amount of precipitate is likely limited by transport of species from the upstream flow. As the size of the simulation domain shrinks, the transport pathway from the inlet decreases as well. This effect correlates with the reactive species concentration distribution as shown in Fig. 8. The concentration gradually decreases downstream from the inlet due to the consumption of reactive species. For *H/d* = 1.5, the gradient of concentration is lower compared to the *H/d* = 1.1 case. Such observation demonstrates the permeability/porosity changing (reduction of *H/d*) in porous materials that results in reduced transport of species, which in turn leads to the slower precipitation in the whole domain.

**Figure 9:**
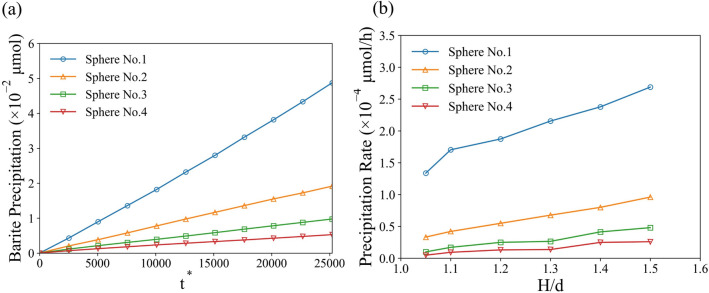
(**a**) Barium sulfate precipitation on each particle as a function of nondimensional time (No. 1–4 left to right). (**b**) Precipitation rate on each particle as a function of *H/d*.

**Figure 10:**
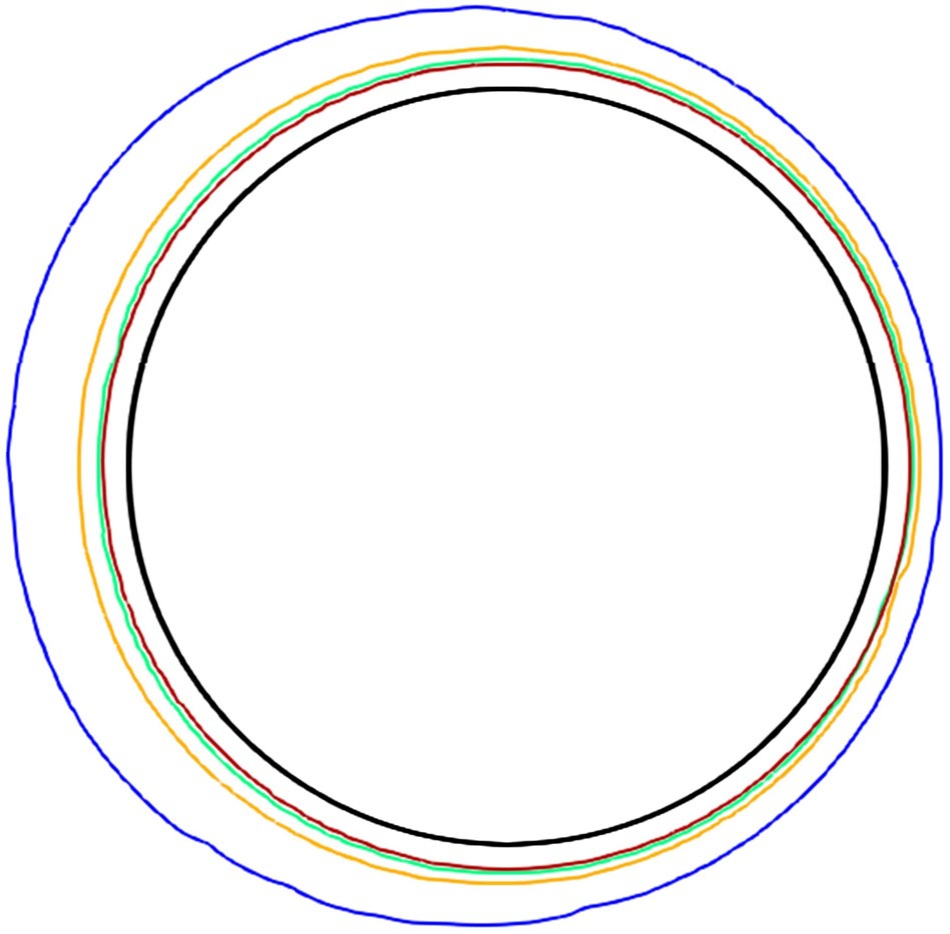
Contour lines of precipitated solid phase (blue: sphere 1, orange: sphere 2, green: sphere 3, red: sphere 4) on spherical particles at 200 h (${t}^{*}$ = 25,200). Black line represents the initial shape of the sphere.

Figure 9a shows the total amount of precipitation on each particle as a function of time. Such result was expected as the Fig. 8 shows that in the vicinity of the front surface of the first spherical particle the reactant concentration was much higher compared to the particles downstream. Therefore, the precipitation on the surface of particles downstream is much slower. Additionally, the simulation shows that the precipitate downstream is more uniformly distributed compared to the first particle. Figure 10 shows the snapshots of solid/liquid interface at 200 h (${t}^{*}$ = 25,200) for different particles. The depletion of the reactant downstream leads to lower reaction rates due to the surface exposure to lower concentrations. As a result, the precipitate layer on the left side is significantly thicker than on the right side for the first particle near the entrance. The precipitation rate on each particle as a function *H/d* is presented in Fig. 9b. As *H/d* increases (porosity increases), the precipitation rate for all particles increases as well. However, the precipitation on the first particle dominates compared to the particles behind it. Due to limitation of scope of this study, we did not explore more complex and realistic pore geometries that include randomized channels and tortuosity. This is a possible topic for the future studies.

### Reaction and transport rates

To demonstrate the capability of the mpFoam solver we chose to simulate the precipitation on a sphere under different flow and chemical reaction conditions. The simulation setup was identical to the validation case, i.e., a 100 $\upmu \text{m}$ radius sphere was submerged in a bulk solution with reactive flow (*SI* = 3). To quantify the impacts of flow condition, reaction rate and diffusive transport, the Péclet number ($Pe=\frac{{\stackrel{-}{{\varvec{u}}}}_{0}d}{D}$) and the second (diffusive) Damköhler number ($Da=\frac{{\dot{r}}_{0}d}{D{C}_{0}}$) were adjusted for the precipitation process. The ${\dot{r}}_{0}$ is a reaction rate for Eq. (7) at saturation state calculate at $C={C}_{0}$.

**Figure 11:**
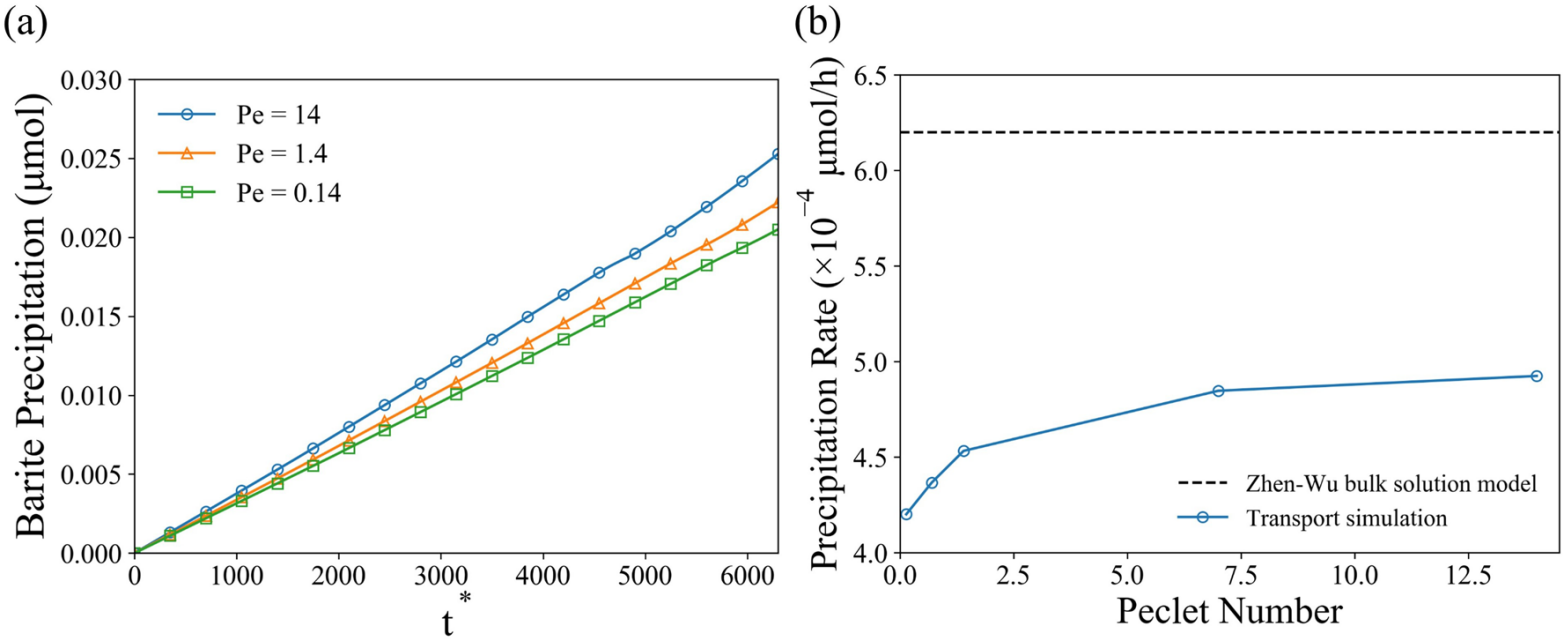
(**a**) Precipitated solid phase mass as a function of nondimensional time for different Péclet number. (**b**) Precipitation rate on particle as a function of Péclet number.

To study the effect of the Péclet number, all other parameters were kept the same as the validation case, while the inlet flow velocity was adjusted. Figure 11a shows the precipitated amount of barite on the sphere surface for $Pe$ = 14, 1.4 and 0.14. The precipitated barite on the sphere surface linearly increases with the *Pe* number. Figure 11b shows the precipitation rate for different $Pe$ number. As the $Pe$ number steadily increases, the convective transport became more and more important supplying the species for reaction near the sphere surface. Hence, the precipitation of barite was accelerated compared to low $Pe$ number case. However, the increase of the flow velocity also requires longer computational time as the time step decreases proportionally due to the Courant–Friedrichs–Lewy number limit, which may cause instability issue if too large. Another observation from the Fig. 11b is that the precipitation rate obtained from simulations were always lower than the rate predicted by the open solution model from experiments (dash line). Such a difference was likely due to fact that the species concentration near the reactive surface was lower than the bulk solution due to a mixture of transport and reaction limitations (depending on the flow condition), which was also observed by Molins et al.^46^ at pore-scale previously while modeling dissolution of calcite. Here we show the same effect during precipitation of a mineral who kinetics are much slower than that of calcite. That there is still a strong influence transport on the kinetics suggests that a “kinetics-limited” regime may be difficult to achieve for minerals of geochemical interest in porous media.

**Figure 12:**
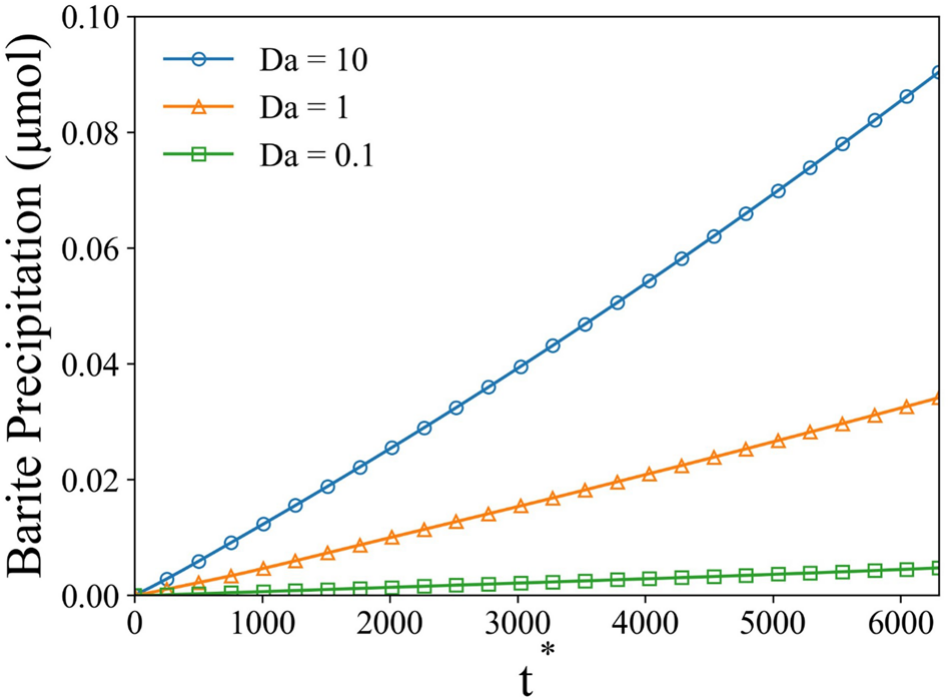
Precipitated solid phase mass as a function of nondimensional time for different Damköhler number at Pe = 1.4.

The second Damköhler number was changed by adjusting the reaction rate for the precipitation. The flow speed was kept at 10 $\upmu \text{m}/\text{s}$ to constrain the convection effect. As the $Da$ number increased, the total amount of precipitated barite increased as well (see Fig. 12). Importantly, the growth of precipitated mass was not directly proportional to the $Da$ number. For instance, as the $Da$ number increased by tenfold from 1 to 10, the total amount of precipitated mass increased by ~ 3.5 times. Such observation is likely due to the limitation of species transport, which in turn reduces the local concentration and suppresses the precipitation process.

**Figure 13:**
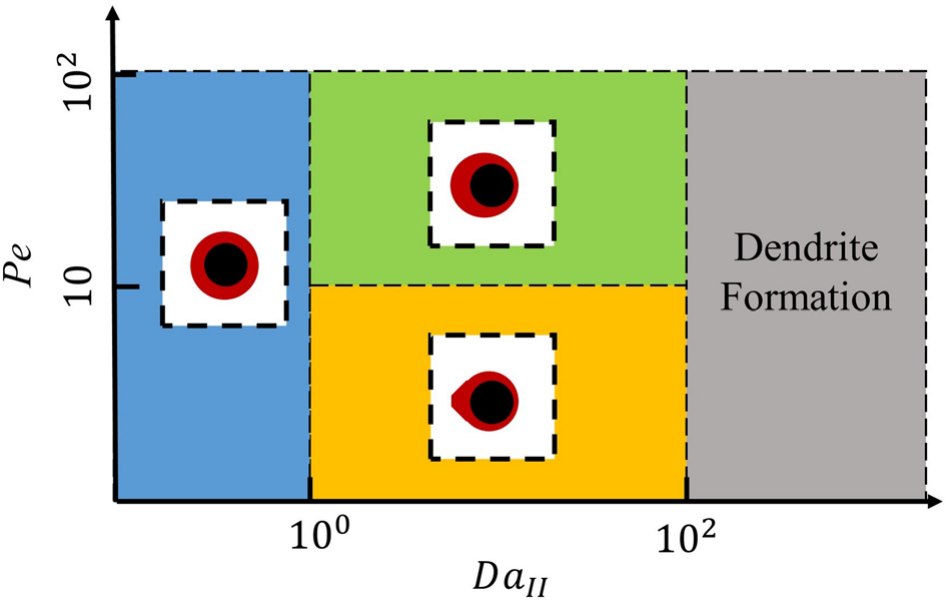
Regime diagram according to Péclet and Damköhler numbers.

Figure 13 shows the diagram of precipitation regimes on a sphere in respect to Péclet and Damköhler numbers. It was observed that, for $Da$ < 1, the precipitation on the sphere was more uniformly distributed due to the abundant transport of species and limited reaction. As the reaction rate and $Da$ number increase, the precipitation was more limited by the diffusion, which resulted in the asymmetry distribution of solid phase. Such observation is similar to previous studies on dissolution of solid phase^24,27^. At higher flow velocities ($Pe$ > 10), the solid phase was formed in an ellipsoid shape as the left side of the sphere had more precipitation compared to right side of the sphere due to heterogeneous distribution of concentration from advection (see Fig. 8). At lower flow velocities ($Pe<10$), the advection of species could not outweigh the reaction on solid surface, which resulted in an elongated shape of solid phase facing the flow direction. This was likely due to combination of limited advection and diffusion compared to reaction, which was observed by Soulaine et al.^24^ in dissolution process. One thing that is different from the dissolution process in precipitation is that, for extremely high $Da$ number, dendrite formation would be observed^47^. However, such a scenario is significantly more complex and requires very detailed studies in the future.

### Activity correction to the molecular diffusion

**Figure 14:**
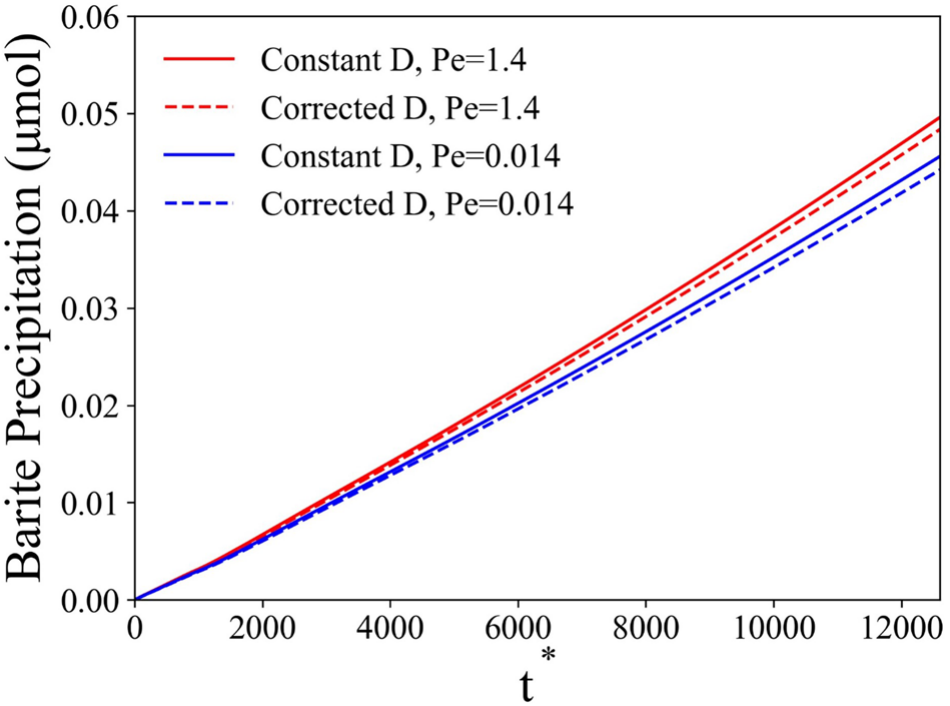
Precipitated solid phase mass as a function of nondimensional time with/without activity correction.

**Figure 15:**
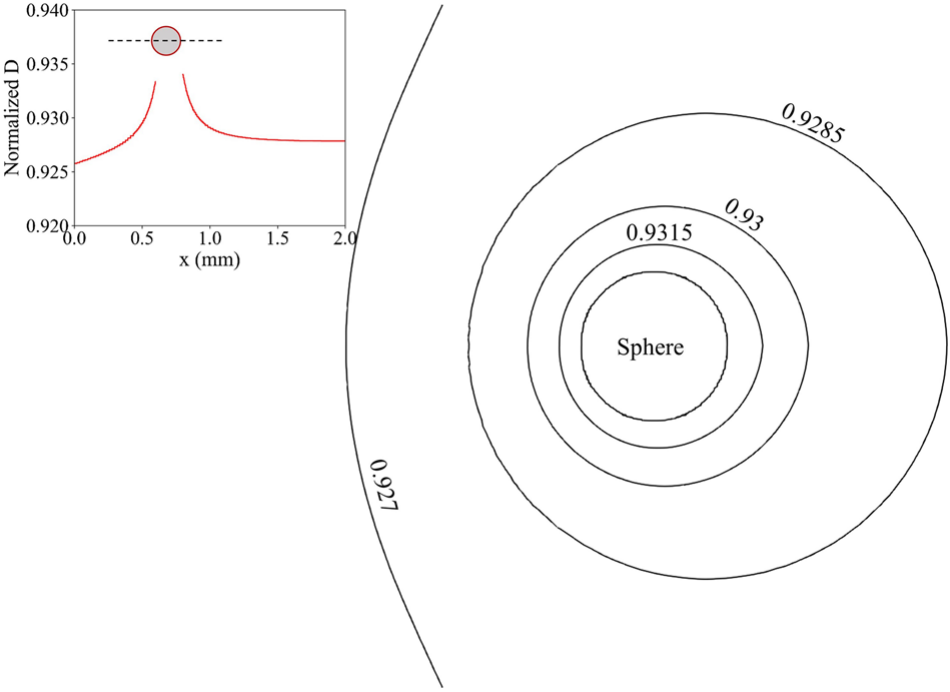
Normalized diffusion coefficient ($D/{D}_{0}$) contour lines around a precipitating particle (insert: Normalized diffusion coefficient along the center line of the particle).

In this section, we study the effect of activity correction to the molecular diffusion on the precipitation process. The theory for activity correction was introduced in “Method” section. Similar to Dutka et al.^42^, the coefficient *A* was obtained from the low-concentration limit of the activity coefficient, while the coefficient *B* was a fitting parameter from the PHREEQC^48^ data for barium sulfate. In our study, the coefficient *A* is 0.02984 and the coefficient *B* is 0.0096 from fitting PHREEQC data. Using the above activity correction to the diffusion coefficient, we modeled precipitation on the spherical particle (${u}_{0}$ = $10\;\upmu \text{m}/\text{s}$). Figure 14 shows the comparison between simulations with a constant diffusion coefficient and activity corrected diffusion coefficient. As the results have shown, the activity correction only has a moderate effect on the total amount of precipitated solid mass in case of barite precipitation. Comparing to the constant diffusion coefficient case, the precipitated solid mass is about 3% lower at 100 h. Further reduce the velocity (${u}_{0}$ = $0.1\;\upmu \text{m}/\text{s}$, blue lines in Fig. 14) does not greatly affect the results either. In general, the diffusion coefficient with activity correction decreased by ~ 7% compared to the diffusion coefficient in bulk. This reflects that the precipitation process is significantly reaction limited. Figure 15 shows the normalized diffusion coefficient ($D/{D}_{0}$) contour lines around the spherical particle. The increase in diffusion coefficient towards the surface is caused by the consumption of reactive species during heterogeneous precipitation. In general, the activity correction of the diffusion coefficient was not significant enough to affect the precipitation process significantly in the current setup.

### Implications for mineral crystal growth modeling

**Figure 16:**
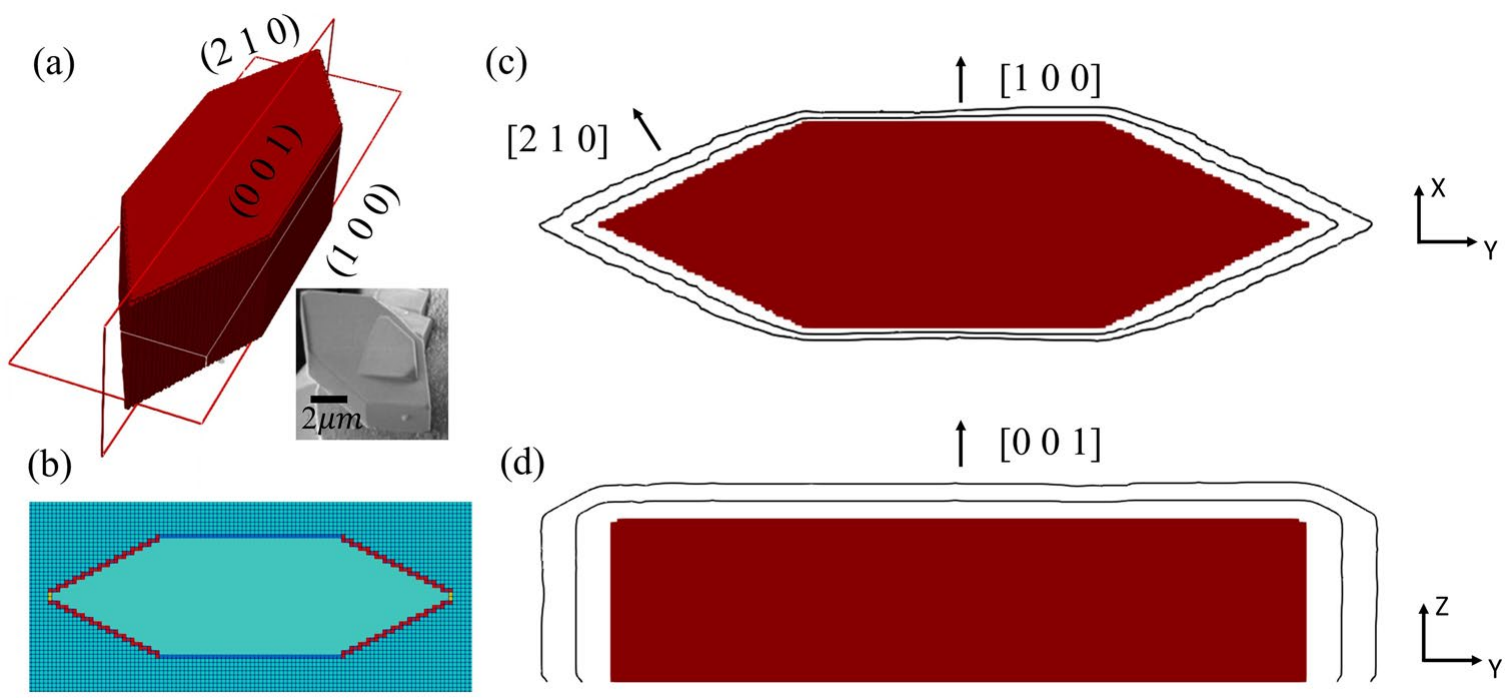
(**a**) Geometry of a barite crystal from experiment. (**b**) Schematics of reaction rate constants assigned to the surfaces of the crystal with different surface norm. (**c**) Contour lines of crystal surface at 3 h and 6 h on the *XY* plate. (**d**) Contour lines of crystal surface at 3 h and 6 h on the *YZ* plate.

The goal of this part of the study is to demonstrate the capability of the developed solver on maintaining the shape of the crystal during growth while its surfaces have different growth rate, which was not studied using DBS approach in the past. The values of $k$ were fitted following the linear kinetic equation, $D\nabla C\cdot {\varvec{n}}={k}_{i}\left({C}_{i}-{C}_{eq}\right)$, from barite crystal growth experiments^33^. Figure 16a shows the shape and surface directional norms obtained from experimental observation^33^. Three different crystallographic orientations ((100), (001), (210)) were considered for this simulation and their surface propagation rates by direction were [100] 30 ± 10 nm/h, [001] 60 ± 10 nm/h, [210] 90 ± 30 nm/h, respectively. The values of reaction rate constants obtained from surface propagation rates were ${k}_{100}$ = 1.442 $\times$ 10^–6^ $\text{m}/\text{s}$, ${k}_{001}$ = 2.884 $\times$ 10^–6^ $\text{m}/\text{s}$, ${k}_{210}$ = 4.325 $\times$ 10^–6^ $\text{m}/\text{s}$. Figure 16b illustrates different reaction rate constants assigned to the different surfaces basing on their surface norms by the solver (red for (210), blue for (100)). The transport of species was controlled by diffusion from bulk, which follows Eq. (6) with $\stackrel{-}{{\varvec{u}}}$ = 0.

The shape of the growing crystal was plotted as contours for the *XY* and *ZY* plates, which were marked out in Fig. 16a. Figure 16c shows the solid–liquid interface contours at 3 h and 6 h on the *XY* plate. As the results show, the (210) surface has a faster growth rate than the (100) surface and the tip sharpness pointing to the (010) was preserved quiet well. Figure 16d shows similar contours for the *ZY* plate. The rates of surface propagation rates obtained from simulation were [100] 31 nm/h, [001] 63 nm/h, [210] 93 nm/h, which means that the model maintains the propagation of the surface well using surface reaction rate constant as a parameter. Overall, the mpFoam solver can capture the directional growth while maintain the general shape of the crystal. However, it has resulted in rounding at the edges of some corners of the crystal (Fig. 16d). The DBS model shows that it is capable of maintains the shape of the crystal during growth, while including different growth rate for surfaces from experiments. Future work will be directed towards investigating the crystal edge evolution. This may reveal the average lifetime of fast-growing facets and kinetics of crystal morphology evolution in general.

## Summary

In this study, we developed a multiphase micro-continuum solver (mpFoam) based on the Darcy–Brinkman–Stokes equation. The solver was implemented using an open-source software package—OpenFOAM. We demonstrated solver’s capability to capture the propagation of the solid interface enhancing this capability by an adaptive mesh refinement. This solver was validated against analytical solution and currently existing ALE solver. The evolution of solid phase interface shows a good agreement with both the analytical solution and ALE solver. This validation demonstrates that the developed solver is capable of simulating the precipitation process of mineral at pore scale. Also, we have shown that if the sharp interface criterion ${\varepsilon }_{s,c}$ was too low, the interface could be diffusive (occupies several layers of cells) and deviate from the analytical solution.

We used the solver to investigate the precipitation process of barite mineral on solid sphere surfaces under different conditions. First, the effect of geometrical constrain (reduced porosity) was studied on spheres arranged as an array with a cubic symmetry. The results have shown that, as the simulation domain shrinks (lower porosity), the total precipitation of barium sulfate decreases. Second, the interplay between the transport and reaction rate were studied in a case of precipitation on a single spherical particle by varying dimensionless *Pe* and *Da* numbers. The results show that the increase in convective over diffusive transport (increasing *Pe*) enhances precipitation, however, not significantly. Contrastingly, the increase in reaction rate over the diffusive transport (increasing *Da*) resulted in significant acceleration of precipitate growth. We conclude that the flow conditions are not important in such regimes and the defining processes are happening in the interfacial layer where the diffusive flux is balanced with the reactive flux. In addition, we implemented an activity correction for the diffusion coefficient based on extended Debye-Hückel theory. The comparison of precipitation rates suggested that only moderate reduction of precipitation (~ 3%) was observed. However, under longer time-scales, such effects are critical to include. Lastly, the directional growth of different crystal planes was implemented using anisotropic growth rates. Growth of a single barite crystal was simulated to test this capability of our solver. Resulting evolution of crystal facets reproduced our previous SEM observations maintaining the shape of the crystal. This demonstration shows the potential for future studies and development of more robust macroscopic precipitation models that take into account microscopic/atomistic phenomena.

The versatility of such a DBS model coupled with adaptive mesh method opens a way for broader applications where the motion of the interface is driven by a surface reaction or a phase transformation. However, to make the model more realistic and suitable for studying a broader range of reactant concentrations, other features such as nucleation need to be introduced. It is also expected that future studies including more realistic pore geometries may improve our understanding of the precipitation process at pore- and meso-scales.

## Supplementary Information

Supplementary Information.
