# Supplementary material for: Micro-continuum approach for mineral precipitation
Source: Sci Rep. 2021 Feb 10;11:3495. doi: 10.1038/s41598-021-82807-y (PMC7876130; doi:10.1038/s41598-021-82807-y)
Supplement: Supplementary file 1 — Supplementary Information. [file 41598_2021_82807_MOESM1_ESM.pdf]

Micro-continuum Approach for Mineral Precipitation

Fengchang Yang*, Andrew G. Stack, Vitalii Starchenko*

Chemical Sciences Division, Oak Ridge National Laboratory, 1 Bethel Valley Rd., Oak Ridge, TN 37831, United States

*email: Fengchang Yang yangf@ornl.gov; Vitalii Starchenko starchenkov@ornl.gov

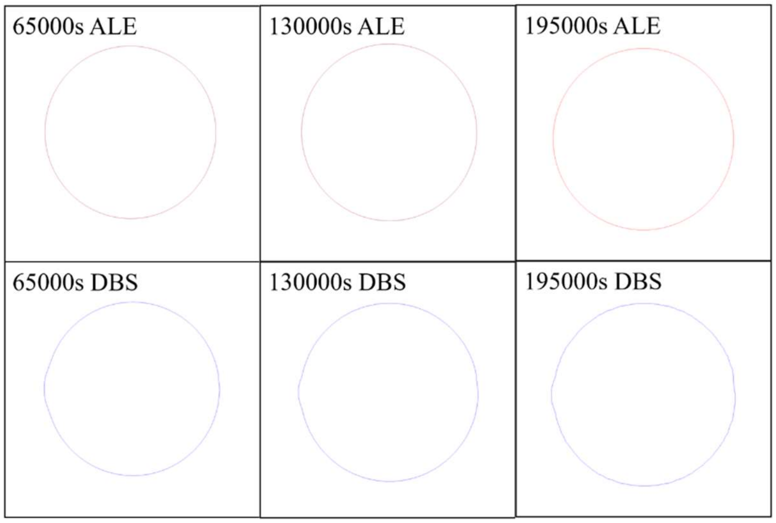

Figure S1. Interface shape comparison between mpFoam and ALE solver.

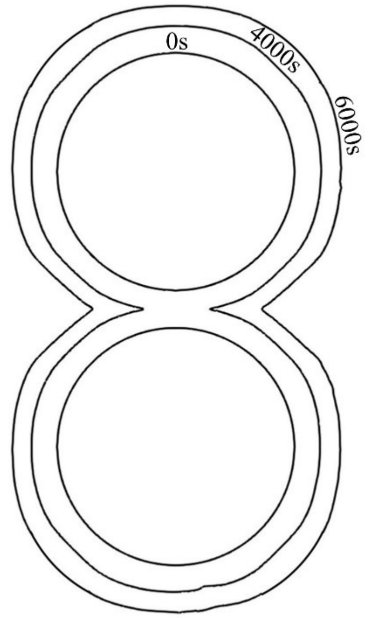

Figure S2. Coalescence of precipitate growing on initially separated spheres.

A simulation was performed to demonstrate the capability of the DBS model on capturing the coalescence of solid phase. Two 100 μm radius spheres were initially placed vertically adjacent to each other with a gap distance of 30 μm. At the inlet, the solution flows at a speed of $\bar{u}_0$=10 $\frac{\mu m}{s}$ and has a concentration of reactant that corresponds to C_0=0.32 $\frac{mol}{m^3}$. The saturation concentration is C_s=0.0105 $\frac{mol}{m^3}$. The precipitation reaction follows Eqn. 7 in the main manuscript and the reaction constant is set as k=2.479×10^{-7} $\frac{mol}{m^2 \cdot s}$. The viscosity of the solution is μ_f=0.89×10^{-6} $\frac{m^2}{s}$ and diffusion coefficient for ions is D=5.0×10^{-7} $\frac{m^2}{s}$. The other simulation parameters are similar to the one performed in the main manuscript (see the geometry of the simulation domain in Fig. 4). Figure S2 shows the evolution of liquid-solid interface as simulation progress, which successfully capture the coalescence of two growing sphere. The two initial separate spheres merged into one solid object due to precipitation.
